# Equine or porcine synovial fluid as a novel *ex vivo* model for the study of bacterial free-floating biofilms that form in human joint infections

**DOI:** 10.1371/journal.pone.0221012

**Published:** 2019-08-15

**Authors:** Jessica M. Gilbertie, Lauren V. Schnabel, Noreen J. Hickok, Megan E. Jacob, Brian P. Conlon, Irving M. Shapiro, Javad Parvizi, Thomas P. Schaer

**Affiliations:** 1 Department of Clinical Sciences, College of Veterinary Medicine, North Carolina State University, Raleigh, NC, United States of America; 2 Comparative Medicine Institute, North Carolina State University, Raleigh, NC, United States of America; 3 Department of Clinical Studies New Bolton Center, School of Veterinary Medicine, University of Pennsylvania, Kennett Square, PA, United States of America; 4 Department of Orthopedic Surgery, Thomas Jefferson University, Philadelphia, PA, United States of America; 5 Department of Population Health and Pathobiology, College of Veterinary Medicine, North Carolina State University, Raleigh, North Carolina, United States of America; 6 Department of Microbiology and Immunology, University of North Carolina-Chapel Hill, Chapel Hill, NC, United States of America; The University of Jordan School of Pharmacy, JORDAN

## Abstract

Bacterial invasion of synovial joints, as in infectious or septic arthritis, can be difficult to treat in both veterinary and human clinical practice. Biofilms, in the form of free-floating clumps or aggregates, are involved with the pathogenesis of infectious arthritis and periprosthetic joint infection (PJI). Infection of a joint containing an orthopedic implant can additionally complicate these infections due to the presence of adherent biofilms. Because of these biofilm phenotypes, bacteria within these infected joints show increased antimicrobial tolerance even at high antibiotic concentrations. To date, animal models of PJI or infectious arthritis have been limited to small animals such as rodents or rabbits. Small animal models, however, yield limited quantities of synovial fluid making them impractical for *in vitro* research. Herein, we describe the use of *ex vivo* equine and porcine models for the study of synovial fluid induced biofilm aggregate formation and antimicrobial tolerance. We observed *Staphylococcus aureus* and other bacterial pathogens adapt the same biofilm aggregate phenotype with significant antimicrobial tolerance in both equine and porcine synovial fluid, analogous to human synovial fluid. We also demonstrate that enzymatic dispersal of synovial fluid aggregates restores the activity of antimicrobials. Future studies investigating the interaction of bacterial cell surface proteins with host synovial fluid proteins can be readily carried out in equine or porcine *ex vivo* models to identify novel drug targets for treatment of prevention of these difficult to treat infectious diseases.

## Introduction

Infectious or septic arthritis is an orthopedic emergency that results in substantial morbidity and mortality[1–3]. *Staphylococcus aureus* (*S*. *aureus*) is the most common bacterial organism isolated from infectious arthritis and also in periprosthetic joint infection (PJI), accounting for the highest treatment failure rates[2,4–7]. These high treatment failure rates are linked to the ability of Staphylococci to form robust biofilms[7–10]. The traditional definition of a biofilm is a community of bacteria within a polymeric matrix that is attached to an abiotic or biotic surface[11]. However, recent advancements in biofilm research suggests that bacteria do not need a surface for formation; rather bacteria may attach to one another or host-derived proteins to form a biofilm[12–16].

Current work in the infectious arthritis field has shown the ability of *S*. *aureus* to form free-floating biofilms in human synovial fluid both *in vitro* and *in vivo*[17–20]. Within that body of work, the authors evaluated both *S*. *aureus* laboratory strains and clinical isolates from human patients [17]. In addition, biofilm aggregates have been described in other locations within the body such as the lungs of cystic fibrosis (CF) patients, the middle ear, and on the skin[12,14,21,22]. These biofilm aggregates displayed antimicrobial tolerance to cefazolin and vancomycin *in vitro*[17–19]. The antimicrobial tolerance displayed by synovial fluid biofilm aggregates is similar to traditional biofilms and the biofilm aggregates that form the sputum of CF patients[13,23,24]. Continued *in vitro* investigations are critical for understanding this novel, free-floating bacterial phenotype in synovial fluid; however, these research efforts can be hampered as they rely on large volumes of synovial fluid which are difficult to source. Moreover, synovial fluid from human donors can be of variable quality due to underlying donor pathology.

To date, rodent and rabbit models have been at the forefront of infectious arthritis and PJI *in vivo* research[25,26]. However, rodent and rabbit cartilage biology as well as inflammatory responses are significantly different from those of humans[27–30]. Moreover, synovial fluid is difficult to obtain in large quantities from these species[31,32].

Large animals such as horses, pigs, goats, sheep and dogs have been successfully used to explore mechanisms of non-infectious joint disease, particularly osteoarthritis[33–35]. The advantage of large animal models is that their cartilage biology is more similar to that of humans than rodents and rabbits[33,36–38] and substantially larger volumes of synovial fluid can be collected. Of all the large animal models, horses and pigs are most commonly used for the study of osteoarthritis because cartilage thickness and response to injury, as well as their overall immune response, is similar to that of humans [33,35,39–41].

The objective of this study was to investigate if equine and porcine synovial fluid can be used as an *ex vivo* model system of human joint infection and to investigate how microbial-synovial fluid interactions limit antimicrobial activity. To achieve this goal, we first investigated whether synovial fluid induced aggregate formation would occur across the aforementioned species. Next, we asked if biofilm aggregate formation would also be observed with non-Staphylococcal species, i.e. *Escherichia coli* (*E*. *coli*), *Streptococcus equi* subspecies *zooepidemicus* (*S*. *zooepidemicus*) and *Pseudomonas aeruginosa* (*P*. *aeruginosa*). We then determined if biofilm aggregate formation in synovial fluid leads to antimicrobial tolerance as a function of antimicrobial class, bacterial species and synovial fluid source. Finally, we asked if enzymatic dispersal of biofilm aggregates could restore antimicrobial activity.

## Results

### *Staphylococcus aureus* forms free-floating biofilm aggregates in equine, human and porcine synovial fluid

**Fig 1:**
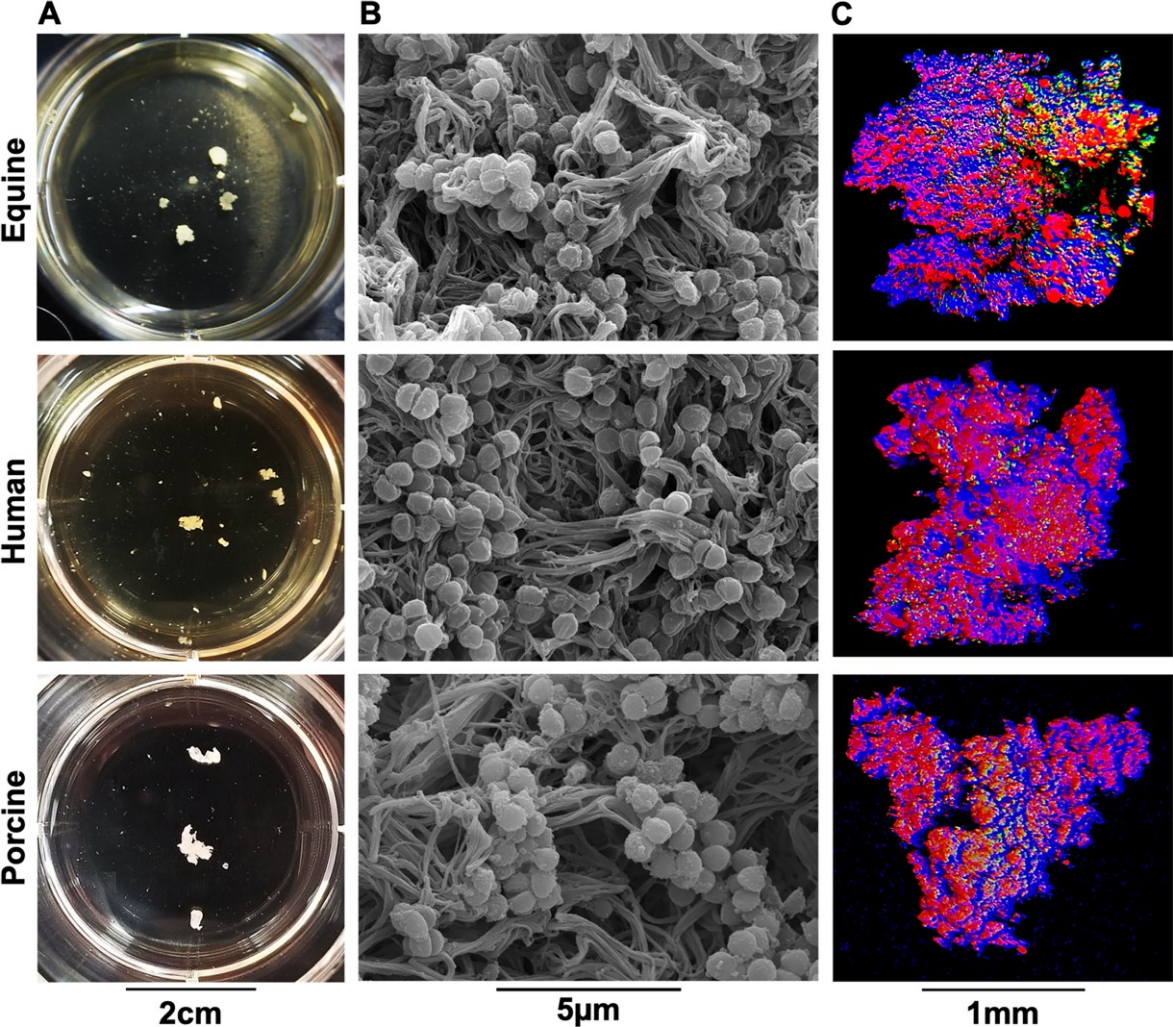
*Staphylococcus aureus* forms macroscopic biofilm aggregates in the synovial fluid of several different species. Equine, human or porcine synovial fluid was infected at 1x10^6^ CFU/mL with *S*. *aureus* (ATCC25923) and incubated overnight at 37°C in a microaerophilic chamber on a shaker at 120rpm to mimic the joint environment. (A) Macroscopic biofilm aggregates were observed in synovial fluid in all three species and photographed. (B) Aggregates were removed from the synovial fluid, fixed, dehydrated in ethanol, sputter coated and imaged with a scanning electron microscope with a FEI-Tecnai T12 microscope showing bacteria nested within a polymeric cord-like extracellular matrix. (C) Aggregates were stained with wheat germ agglutinin (WGA (blue)) for carbohydrates, SYTO9 for nucleic acids/bacteria (green), and SYPRO (red) for proteinaceous content. Confocal laser scanning microscopy (CLSM) was performed using a 12.5x upright lens on a Leica SP5 Multiphoton Microscope. CLSM images were generated as 3-D reconstructions by sequential Z-stacking and tile scanning with Velocity software.

When grown in human or bovine synovial fluid, *S*. *aureus* or *S*. *epidermidis*, respectively, formed free-floating biofilm aggregates[17,20]. We first ascertained if *S*. *aureus* could form biofilm aggregates in equine and porcine synovial fluid with similar structure to those formed in human synovial fluid. Upon incubation of synovial fluid with *S*. *aureus*, aggregation was macroscopically evident within ~ 1–2 hours post-infection and biofilm aggregate size reached a plateau at ~16–18 hours post infection (S1 Fig). We found that *S*. *aureus* formed biofilm aggregates in all species by 24 hours post-infection (Fig 1A). Analysis using scanning electron microscopy (SEM) indicated that biofilm aggregates had similar ultrastructure in equine, porcine and human synovial fluid. In each species, we observed *S*. *aureus* contained within a polymeric, cord-like, extracellular matrix (Fig 1B). Using confocal microscopy three-dimensional (3D) reconstruction, we observed that synovial fluid biofilm aggregates exhibited a mixed protein (red, SYPRO) and carbohydrate (blue, WGA) extracellular matrix; nucleic acid/bacterial staining (green, SYTO9) was scattered throughout the aggregate in all three species (Fig 1C).

### Non-Staphylococcal arthrotropic bacteria form biofilm aggregates in equine synovial fluid

**Fig 2:**
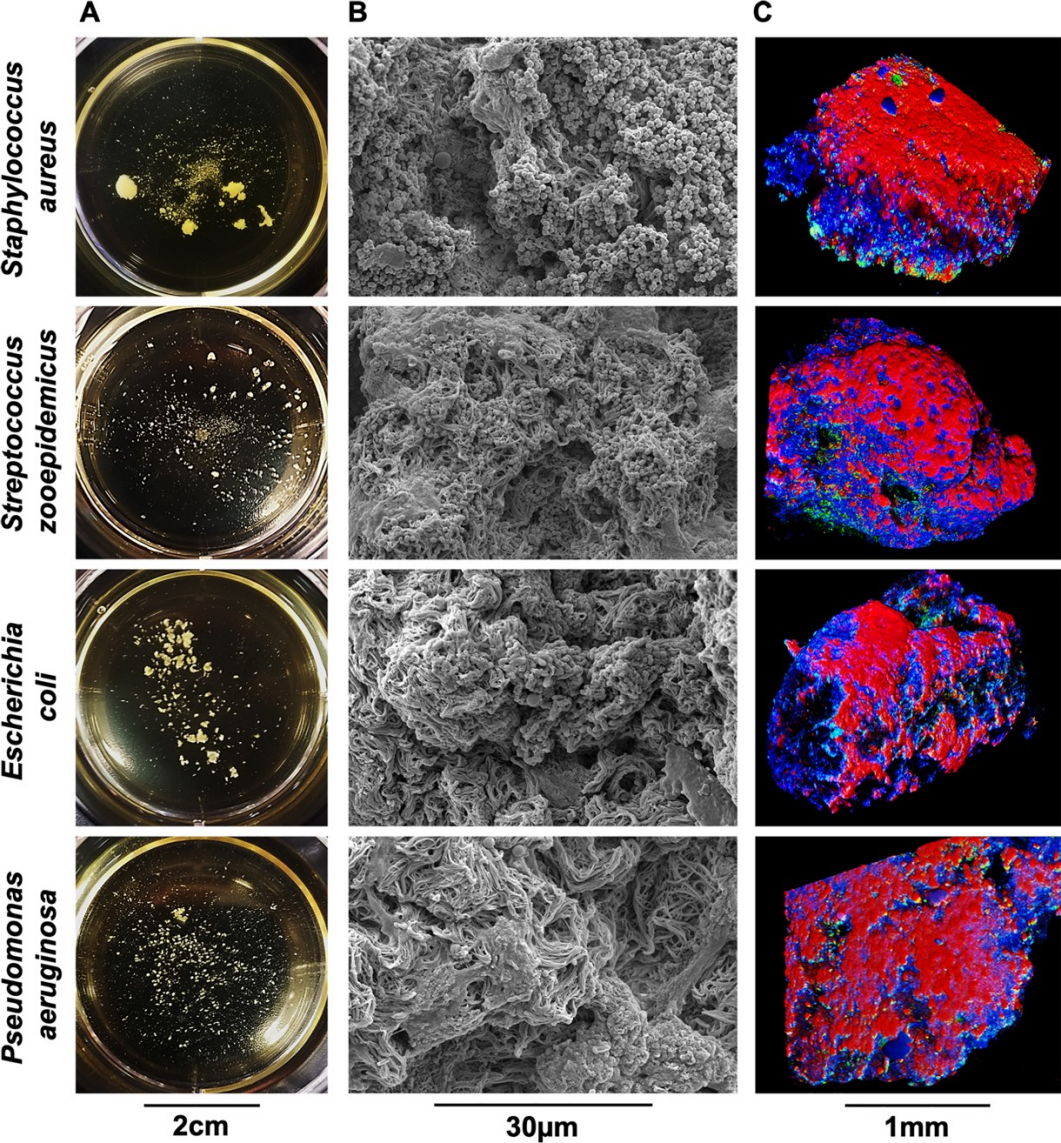
Gram-positive and gram-negative arthrotropic clinical isolates form macroscopic biofilm aggregates in equine synovial fluid. Equine synovial fluid was infected at 1x10^6^ CFU/mL for each clinical isolate (*S*. *aureus*, *S*. *zooepidemicus*, *E*. *coli*, and *P*. *aeruginosa*) and incubated overnight at 37°C in a microaerophilic chamber on a shaker at 120rpm to mimic the joint environment. (A) Macroscopic bacterial aggregates were observed in synovial fluid for all four strains and photographed. (B) Aggregates visualized by SEM as in Fig 1. (C) Aggregates visualized by confocal microscopy using WGA, Syto9 and SYPRO as in Fig 1.

Dastgheyb et al.[17] observed that methicillin-resistant (MRSA) and methicillin-sensitive *S*. *aureus*, from both laboratory-adapted strains and clinical isolates from cases of human septic arthritis, formed biofilm aggregates in synovial fluid. Therefore, we next asked if the biofilm aggregate phenotype that develops in synovial fluid was restricted to *S*. *aureus*. Using arthrotropic clinical isolates derived from equine septic arthritis cases, we infected synovial fluid with *S*. *aureus*, *S*. *zooepidemicus*, *E*. *coli*, or *P*. *aeruginosa*. These strains represent the most common isolates from equine infectious arthritis cases seen at the University of Pennsylvania George D. Widener Hospital Large Animal Hospital in the last five years[42]. By 24 hours, *S*. *aureus* formed large biofilm aggregates; *S*. *zooepidemicus*, *E*. *coli*, and *P*. *aeruginosa* also formed aggregates in synovial fluid, although the aggregates were smaller than those formed with *S*. *aureus* (Fig 2A). Micrograph analysis revealed that all biofilm aggregates were comprised of a polymeric, cord-like, extracellular matrix heavily colonized by bacteria whose morphology and size was consistent with bacterial strain (Fig 2B). Confocal microscopy 3D renderings of biofilm aggregates showed similarities between all isolates (compare Fig 2C to Fig 1C).

### Synovial fluid biofilm aggregates display antimicrobial tolerance to several classes of drugs

**Fig 3:**
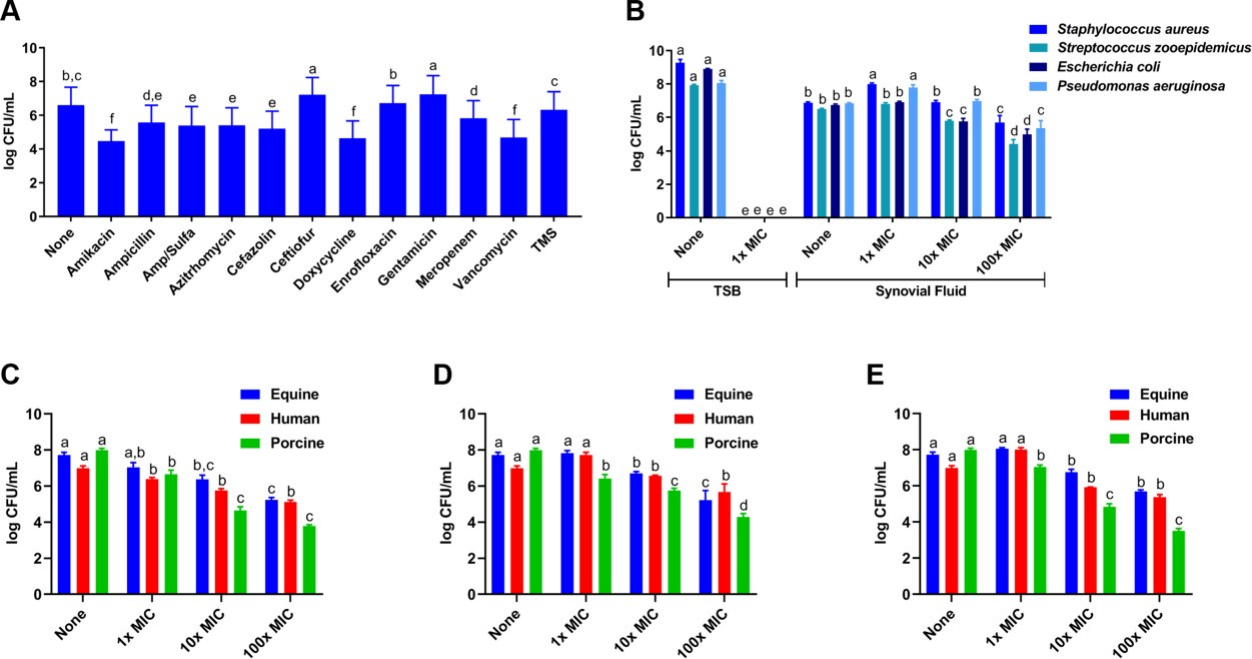
Synovial fluid biofilm aggregates show antimicrobial tolerance against several different classes of antimicrobials. (A) *S*. *aureus* (ATCC25923) biofilm aggregates were allowed to form in equine synovial fluid for 6 hours and this aggregate-containing synovial fluid was treated with a panel of different antimicrobials from several drug classes at 100× the minimum inhibitory concentration (MIC) as determined by *in vitro* antimicrobial susceptibility testing (Table 1). No concentration of any antimicrobial evaluated in this experiment was able to completely kill *S*. *aureus* grown in synovial fluid. (B) The four arthrotropic bacterial isolates from Fig 2 were grown in equine synovial fluid or in tryptic soy broth (TSB). Synovial fluid or TSB was subsequently challenged with 1×, 10× or 100× MIC amikacin, an antibiotic with broad spectrum activity; these isolates were susceptible to amikacin based on our *in vitro* antimicrobial susceptibility data. (C-E) *S*. *aureus* (ATCC25923) were incubated in equine, human or porcine synovial fluid and this bacterial aggregate-containing synovial fluid was subsequently treated with amikacin (C), doxycycline (D) or vancomycin (E) at 1×, 10× or 100× MIC. Bars are means and standard deviations of four biological replicates (i.e. synovial fluid from four individual horses, humans or pigs; n = 4), and significant differences (p<0.05) as determined by ANOVA with Tukey post-hoc are indicated by differing letters.

We asked whether antimicrobial activity of several classes of antimicrobials against *S*. *aureus* would be altered when cultured in equine synovial fluid (MIC concentrations presented in Table 1). For all antimicrobials, planktonic bacteria in tryptic soy broth (TSB) were inhibited or killed at 100× MIC (S2 Fig). In equine synovial fluid, *S*. *aureus* biofilm aggregates persisted *in vitro* at 100× MIC (Fig 3A). Specifically, amikacin (an aminoglycoside), doxycycline (a tetracycline), and vancomycin (a glycopeptide) reduced *S*. *aureus* bacterial concentration by >2 log CFU/mL in equine synovial fluid (p<0.008; Fig 3A). As all four clinical isolates were susceptible to amikacin (based on antimicrobial susceptibility testing (Table 1)), we then used amikacin at 1×, 10× and 100× MIC to screen antimicrobial activity against biofilm aggregates in synovial fluid formed by these isolates. *S*. *zooepidemicus*, *E*. *coli*, and *P*. *aeruginosa* were also killed by amikacin at 1× MIC when grown in TSB planktonically (p<0.0001; Fig 3B). Little to no killing was seen when the drug was used at 1× or 10× MIC against aggregate-containing synovial fluid (Fig 3B); at 100× MIC, a modest decrease in bacterial concentration (~2–3 log CFU/mL) was observed (p<0.009; Fig 3B). Similarly, these bacteria in synovial fluid from equine, human and porcine sources were tolerant to amikacin (Fig 3C), doxycycline (Fig 3D) and vancomycin (Fig 3E) at 1×, 10× and 100× MIC.

**Table 1:** Median minimum inhibitory concentration of clinical isolates and ATCC25923 measured by antimicrobial susceptibility testing using the Sensititre Complete Automated AST System and the equine (Equine EQUIN1F Vet AST Plate) antimicrobial susceptibility panel.

Antimicrobial	Range	*S*. *aureus* (ATCC 25923)	*S*. *aureus*	*S*. *zooepidemicus*	*E*. *coli*	*P*. *aeruginosa*
**Amikacin**	4–32	≤ 4	≤ 4	8	≤ 4	≤ 4
**Ampicillin**	0.25–32	0.5	≤ 0.25	≤ 0.25	4	≥ 32
**Azithromycin**	0.25–4	0.5	≤ 0.25	≤ 0.25	2	≥ 4
**Cefazolin**	4–16	≤ 4	≤ 4	≤ 4	1	1
**Ceftazidime**	1–64	8	2	≤ 1	16	8
**Ceftiofur**	0.25–4	0.5	0.5	≤ 0.25	0.5	4
**Chloramphenicol**	4–32	8	≤ 4	≤ 4	8	≥ 32
**Clarithromycin**	1–8	≤ 1	≤ 1	≤ 1	8	≥ 8
**Doxycycline**	2–16	≤ 2	≤ 2	≤ 2	≤ 2	≥ 16
**Enrofloxacin**	0.25–2	≤ 0.25	≤ 0.25	1	≤ 0.25	1
**Erythromycin**	0.25–8	0.5	≤ 0.25	≤ 0.25	8	≥ 8
**Gentamicin**	1–8	≤ 1	≤ 1	≤ 1	≤ 1	2
**Imipenem**	1–8	≤ 1	≤ 1	≤ 1	≤ 1	1
**Oxacillin+2% NaCl**	0.25–4	≤ 0.25	≤ 0.25	≤ 0.25	≥ 4	≥ 4
**Penicillin**	0.06–8	≤ 0.06	≤ 0.06	≤ 0.06	≥ 8	≥ 8
**Rifampin**	1–4	≤ 1	≤ 1	≤ 1	≥ 4	≥ 4
**Tetracycline**	2–8	≤ 2	≤ 2	8	4	≥ 8
**Ticarcillin**	8–64	≤ 8	≤ 8	≤ 8	16	16
**Ticarcillin-clavulanate**	8/2-64/2	≤ 8/2	≤ 8/2	≤ 8/2	≤ 8/2	16/2
**Trimethoprim Sulfa**	0.5/9.5-4/76	1/19	≤ 0.5/9.5	≤ 0.5/9.5	≤ 0.5/9.5	4/76

### Enzymatic targeting of the proteinaceous matrix disperses synovial fluid biofilm aggregates

**Fig 4:**
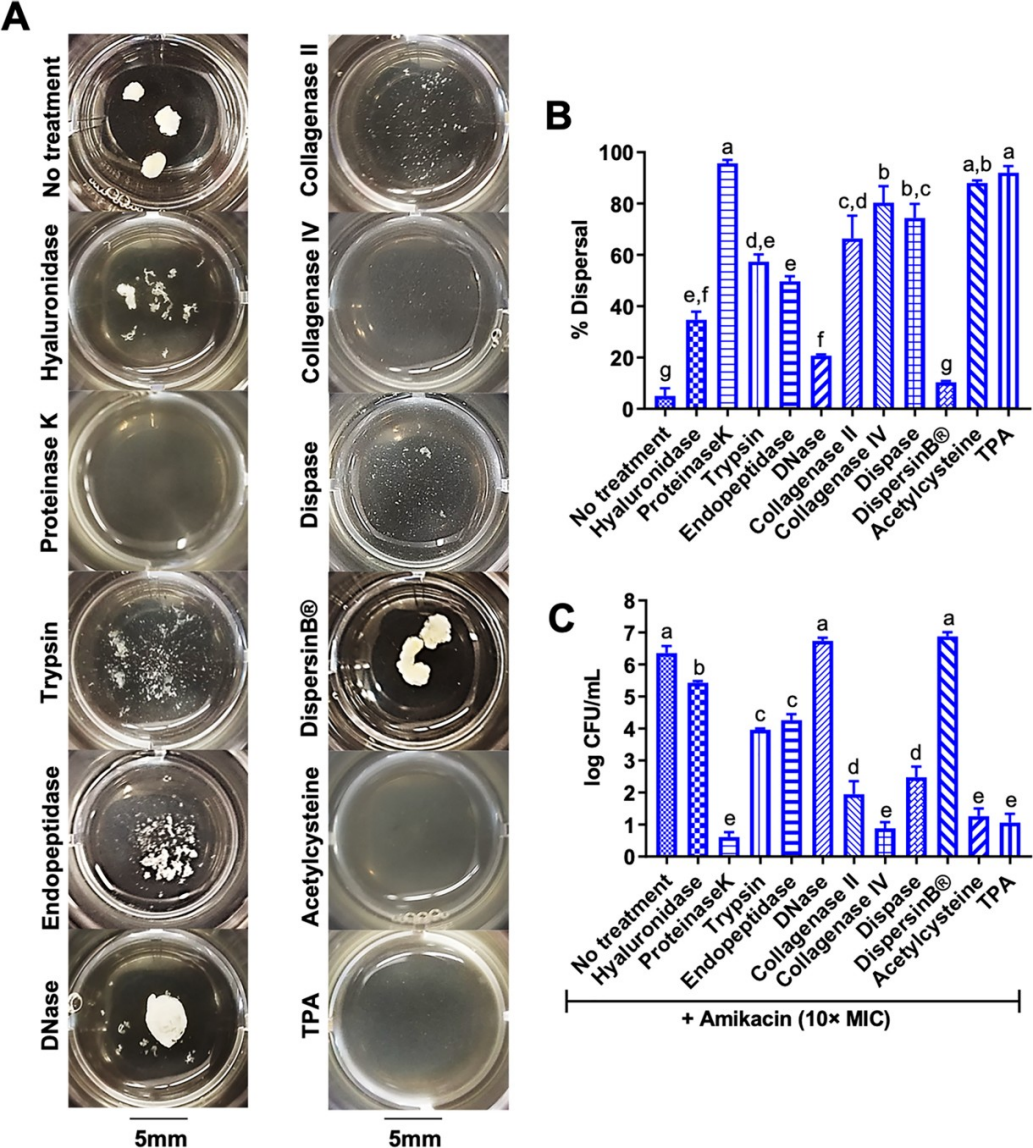
Enzymatic dispersal of synovial fluid biofilm aggregates restores antimicrobial efficacy. (A) *S*. *aureus* (ATCC25923) biofilm aggregates in equine synovial fluid were treated with several enzymes in an attempt to breakdown the extracellular matrix and disperse the bacteria: hyaluronidase (1mg/mL), proteinaseK (200μg/mL), trypsin (200μg/mL), endopeptidase or LysC (200μg/mL), DNase (500μg/mL), collagenase type II (750μg/mL), collagenase type IV (750μg/mL), dispase (500μg/mL), DispersinB (1mg/mL), acetylcysteine (8mg/mL) and tissue plasminogen activator or TPA (1mg/mL). Synovial fluid containing biofilm aggregates was treated with the respective enzyme for 1 hour prior to macroscopic imaging. (B) Percent (%) dispersal was evaluated by measuring absorbance (600nm) and calculating a percentage compared to planktonic *S*. *aureus* grown in TSB to a similar CFU/mL. (C) After 1 hour of dispersion, amikacin was added at 10× MIC (40μg/mL) and log CFU/mL was measured with serial dilutions and colony counting 8 hours post-antimicrobial challenge. Bars are means and standard deviations of four biological replicates (n = 4), and significant differences (p<0.05) as determined by ANOVA with Tukey post-hoc are indicated by differing letters.

Since confocal microscopy showed that synovial fluid biofilm aggregates display a mixed sugar and protein matrix (Figs 1C and 2C) we tested the ability of different enzymes to hydrolyze the synovial fluid biofilm aggregate matrix, disperse the bacteria and improve antimicrobial activity. After *S*. *aureus* aggregate formation in synovial fluid, enzymes including DNase, DispersinB and proteinaseK, among others, were added to infected synovial fluid to target key molecules within the aggregate extracellular matrix. In agreement with Dastgheyb et al.[17], we showed that DispersinB and DNase did not disperse, while proteinaseK was able to completely disperse, *S*. *aureus* biofilm aggregates in synovial fluid (p<0.004; Fig 4A and 4B). Similar to Ibberson et al.[43], pre-treatment of synovial fluid with hyaluronidase, an enzyme that targets hyaluronic acid, the main carbohydrate in synovial fluid, prevented aggregate formation (S3A and S3B Fig); nevertheless, post-treatment with hyaluronidase only mildly dispersed biofilm aggregates in synovial fluid (p<0.02; Fig 4A and 4B). In addition, the proteolytic enzymes trypsin, endopeptidase (LysC), collagenase (type II), and dispase were also able to moderately disperse aggregates in synovial fluid (p<0.001; Fig 4A and 4B). Finally, collagenase (type IV), acetylcysteine and tissue plasminogen activator (TPA) were similar to proteinaseK in that they significantly dispersed aggregates in synovial fluid (p<0.0001; Fig 4A and 4B).

### Enzymatic dispersal of synovial fluid biofilm aggregates restores antimicrobial activity

Several studies have reported that dispersal of biofilms restores the activity of several classes of antimicrobials[18,44–46]. To determine if dispersal of *S*. *aureus* biofilm aggregates restores antimicrobial activity, synovial fluid containing bacterial aggregates was treated with each enzyme for 1 hour, prior to challenge with amikacin at 10× MIC. Bacterial concentration (log CFU/mL) was measured by serial dilutions and plate counting 8 hours post-antimicrobial challenge. Control wells with enzymes alone did not alter bacterial concentration more than 1 log CFU/mL compared to untreated synovial fluid (S4 Fig). Trypsin, endopeptidase (LysC), collagenase (type II) and dispase treatment prior to challenge with amikacin moderately increased antimicrobial killing when compared to synovial fluid (containing aggregates) not treated with enzymes (p<0.01; Fig 4C). ProteinaseK, collagenase (type IV), acetylcysteine and TPA treatment prior to challenge with amikacin markedly increased antimicrobial activity as compared to biofilm aggregates in synovial fluid not treated with enzymes (p<0.0003; Fig 4C). Pre-treatment with hyaluronidase mildly increased antimicrobial killing (p<0.04) while no change was observed with DNase or DispersinB treatment (Fig 4C).

### TPA disperses Staphylococcal and non-Staphylococcal biofilm aggregates and restores antimicrobial activity in synovial fluid from multiple species

**Fig 5:**
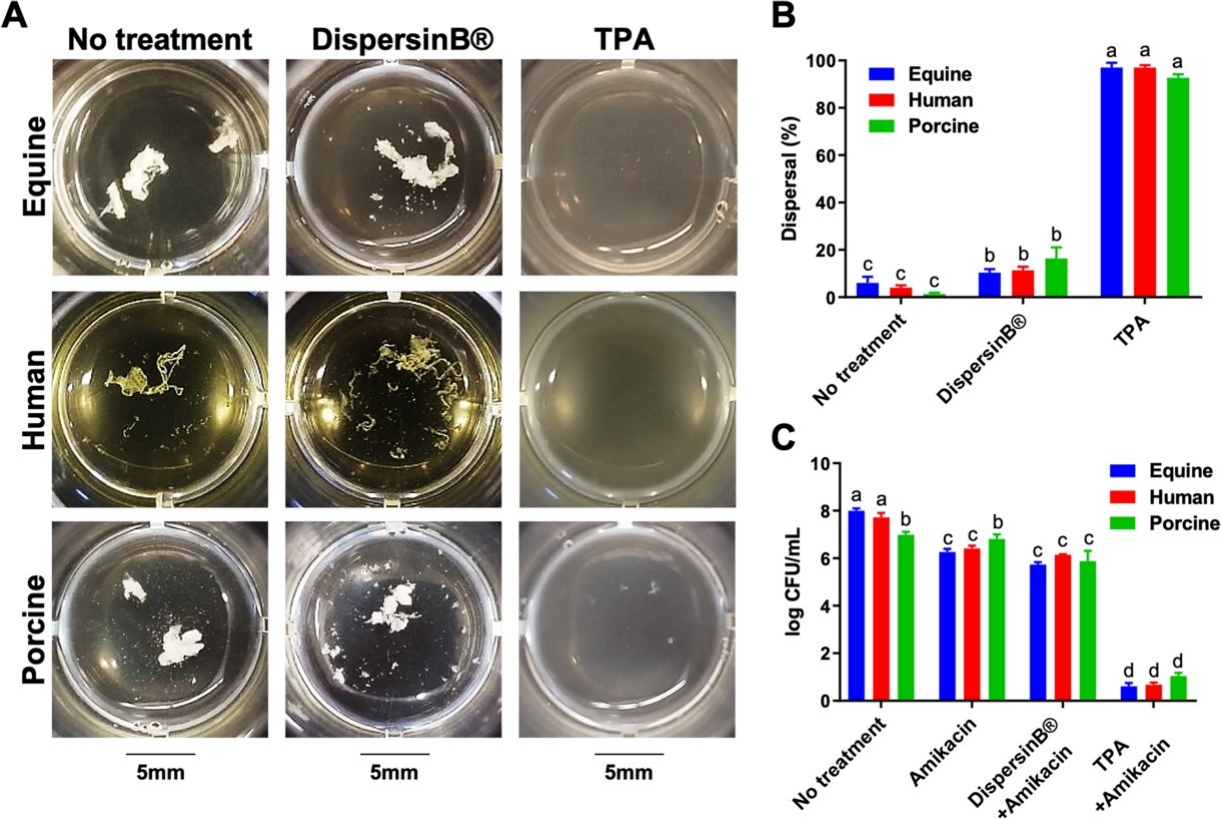
Tissue plasminogen activator (TPA) disperses *S*. *aureus* biofilm aggregates and restores antimicrobial activity in equine, human and porcine synovial fluid. (A) Equine, human and porcine synovial fluid containing *S*. *aureus* (ATCC25923) biofilm aggregates was left untreated or treated with DispersinB (1mg/mL) or TPA (1mg/mL) for 1 hour prior to macroscopic imaging. (B) Percent (%) dispersal was evaluated by measuring absorbance (600nm) and calculating a percentage compared to tryptic soy broth (TSB) containing planktonic *S*. *aureus* at a similar CFU/mL. (C) After 1 hour of dispersion, amikacin was added at 10× MIC (40μg/mL) and log CFU/mL was measured with serial dilutions and colony counting 8 hours post-antimicrobial challenge. Bars are means and standard deviations of four biological replicates (n = 4), and significant differences (p<0.05) as determined by ANOVA with Tukey post-hoc are indicated by differing letters.

**Fig 6:**
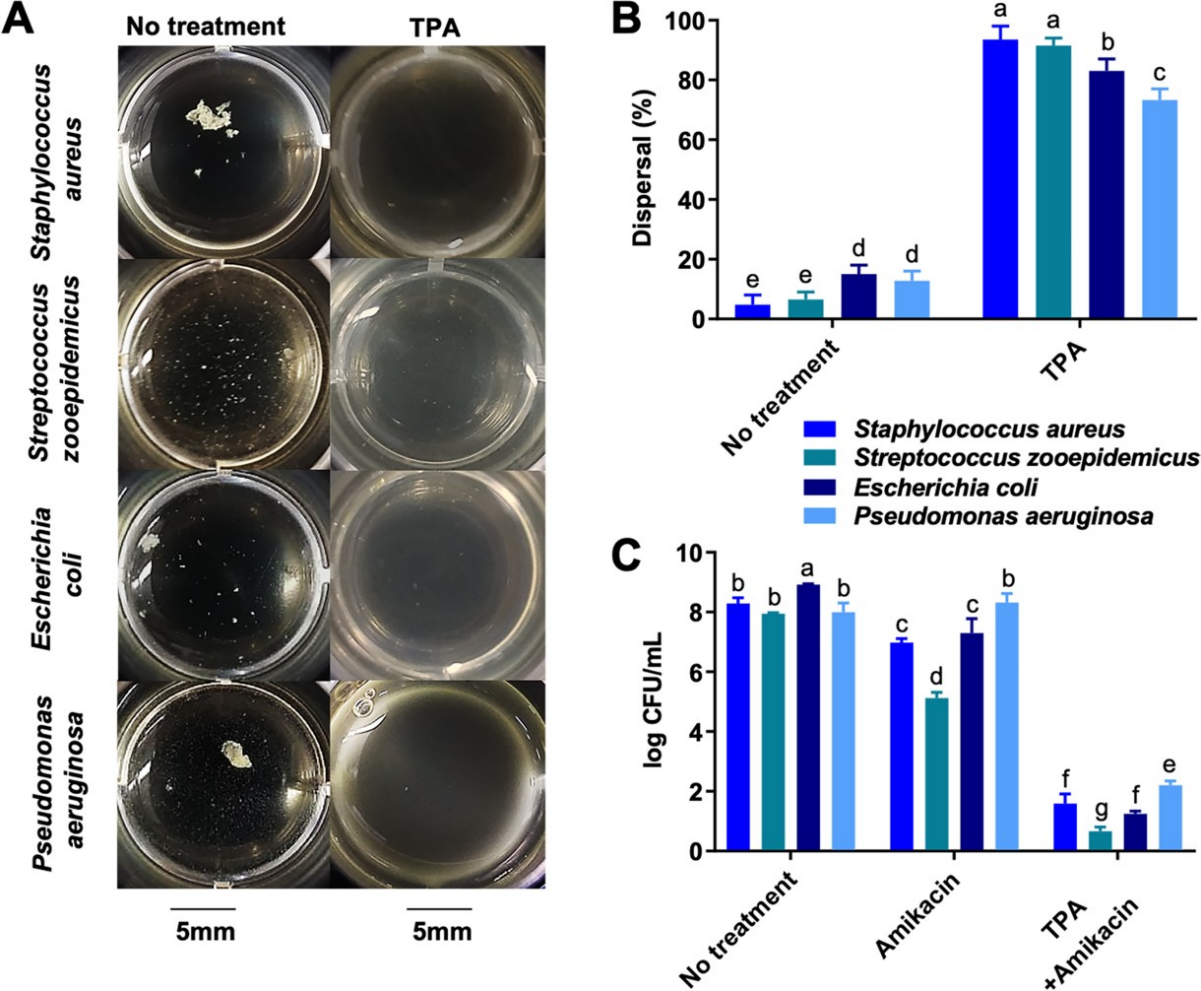
TPA disperses synovial fluid biofilm aggregates and restores antimicrobial activity against both gram-negative and gram-positive aggregates. (A) The four arthrotropic bacterial isolates from Fig 2 were grown in equine synovial fluid. Synovial fluid containing these aggregates bacteria was treated with TPA (1mg/mL) for 1 hour prior to macroscopic imaging. B) Percent (%) dispersal was evaluated by measuring absorbance (600nm) and calculating a percentage compared to planktonic *S*. *aureus* grown in TSB to a similar CFU/mL. (C) After 1 hour of dispersion, amikacin was added at 10× MIC (40μg/mL) to the infected synovial fluid containing biofilm aggregates and log CFU/mL was measured with serial dilutions and colony counting 8 hours post-antimicrobial challenge. Bars are means and standard deviations of four biological replicates (n = 4), and significant differences (p<0.05) as determined by ANOVA with Tukey post-hoc are indicated by differing letters.

Because TPA most effectively dispersed *S*. *aureus* biofilm aggregates in equine synovial fluid (Fig 4A and 4B), we determined if it would also disperse *S*. *aureus* biofilm aggregates in human and porcine synovial fluid. We found that TPA was able to visibly disperse aggregates in equine, human, and porcine synovial fluid (p<0.0002; Fig 5A) and that dispersal was able to restore the antimicrobial activity of amikacin against *S*. *aureus* at 10× MIC (p<0.0004; Fig 5B). In addition, we showed that TPA could disperse aggregates of the equine clinical isolates (*S*. *aureus*, *S*. *zooepidemicus*, *E*. *coli* and *P*. *aeruginosa*) (Fig 6A), with restoration of antimicrobial activity against both gram-positive and gram-negative bacteria cultured in equine synovial fluid (p<0.001; Fig 6B).

## Discussion

The purpose of this study was to examine the ability of *S*. *aureus*, the most commonly isolated bacteria from cases of infectious arthritis and periprosthetic joint infection[2,4–7], as well as non-Staphylococcal species, to aggregate and form free-floating biofilms in equine and porcine synovial fluid, a characteristic of infected human synovial fluid. We provide compelling evidence that biofilm aggregates are similar in structure and function across species indicating that both equine and porcine synovial fluid can be used as *ex vivo* model systems of human joint infection.

Biofilm aggregate formation in synovial fluid offers protection from traditional antimicrobial therapies[17,19] and the host immune system[47–49]. The results presented here show that antimicrobials can be used at 100× MIC in synovial fluid with little to no killing activity *in vitro*, while planktonic cells are easily killed at the same MIC. Based on the wildtype MIC of 4μg/mL used in this study, the clinical breakpoint for susceptible isolates of *S*. *aureus*, *S*. *zooepidemicus*, *E*. *coli*, and *P*. *aeruginosa*, and pharmacokinetic studies in horses[50], the aminoglycoside amikacin can achieve a concentration of ~5× MIC in synovial fluid after intravenous administration of a clinically relevant 10mg/kg dose. However, unlike systemic administration, local administration of amikacin to horses by regional limb perfusion or direct intra-articular injection can achieve a concentration in synovial fluid up to 100× MIC[51–53]. Conversely, local administration of amikacin to inflamed joints decreases the maximal concentration to ~50× MIC[52]. Therefore, concentrations between 1× to 100× MIC are considered within pharmacodynamic range of amikacin. Nevertheless, our studies show that we could not achieve a significant antibacterial effect even at 100× MIC of amikacin against bacteria cultured in synovial fluid due to their aggregation. These results indicate that clinical dosing of amikacin would be ineffective against biofilm aggregates in synovial fluid, which offers a possible explanation for persistent sepsis and increased severity of degenerative joint disease following infectious arthritis[54,55].

The antimicrobial tolerance displayed by bacteria in synovial fluid is similar to surface-attached biofilms and other biofilm aggregates such as those of *P*. *aeruginosa* in the sputum of CF patients[13,23,24]. *In vitro* models of *P*. *aeruginosa* aggregation in synthetic sputum or on alginate beads imparts significant antimicrobial tolerance[56–59], similar to that observed in this *in vitro* infectious arthritis model.

New methods to evaluate the pharmacodynamics of antimicrobial agents within biofilms are being developed[24,45]. One such method is called a minimum biofilm eradication concentration (MBEC), which is the biofilm equivalent to a planktonic minimum bactericidal concentration (MBC)[60]. The *S*. *aureus* MBC for aminoglycosides is within 1× to 4× MIC whereas the MBEC for aminoglycosides can reach concentrations greater than 1000× the MIC/MBC[61–63]. The MIC and MBC for amikacin can be achieved using clinical doses; however, the MBEC concentration is not within the therapeutic index of aminoglycosides. For example, concentrations higher than those currently achieved by local administration of amikacin are toxic to the articular cartilage and can cause nephrotoxicity or ototoxicity by systemic administration[64,65]. These observations correlate with pharmacokinetic studies reporting that MBECs are typically not achievable using clinical doses within the planktonic therapeutic index[66–68]. Therefore, new methods that combine tissue location-specific pharmacokinetic data and biofilm-specific pharmacodynamic data are vital to improve the clinical treatment of biofilm infections. The methods developed in this study could serve as a platform with improved translational fidelity to generate *in vitro* pharmacodynamics data within the articular-specific location.

In this study, we show dispersal of synovial fluid biofilm aggregates restores antimicrobial activity. This is similar to other reports of restoration of antimicrobial activity after dispersing surface-attached biofilms *in vitro* and *in vivo*[18,44–46]. Most biofilm *in vitro* studies rely heavily on traditional microbiological media, such as tryptic soy broth (TSB), which yields a biofilm matrix composed of bacterial-derived polysaccharide intercellular adhesion (PIA)[69–71]. In that regard, DispersinB, an enzyme that specifically targets PIA, has the ability to disperse *S*. *aureus* biofilms formed *in vitro* by traditional methods, whereas proteinaseK is unable to do so[72]. In contrast, this report shows that the biofilm aggregate matrix generated in synovial fluid is predominantly composed of proteins; therefore, treatment with proteinaseK, among other enzymes with proteolytic activity, but not DispersinB, dispersed aggregates. These results line up with previous work that reported PIA-independent biofilm aggregate formation in synovial fluid and dispersal with proteinaseK[17,43] In addition, other studies have noted that *in vivo* biofilms and biofilm aggregates tend to be embedded in a host-derived extracellular matrix versus a bacterial self-produced matrix such as PIA[12]. *S*. *aureus* in particular has several mechanisms to hijack host fibrinogen[48,73]. Since TPA was able to disperse synovial fluid biofilm aggregates, further investigation into the role of fibrinogen as an extracellular matrix component is warranted. Due to the ability of dispersion to restore antimicrobial activity, dispersal agents could decrease the MBEC of biofilm aggregates to fall within the therapeutic index of clinically relevant antimicrobial agents. This is a promising area of future study.

Human synovial fluid, particularly non-diseased, is difficult to obtain and identification of an alternative model that allows for movement between *in vitro* and *in vivo* components is critically needed to advance the field of biofilm aggregate research. Our findings show that both equine and porcine synovial fluid allow for robust biofilm aggregate formation with similar phenotypes to biofilm aggregates formed in human synovial fluid. Noteworthy, although rodent and rabbit models have been used extensively in infectious arthritis and PJI *in vivo* research[25,26,74], it is impractical to use these species for the *ex vivo* studies we have described here. The volume of synovial fluid able to be obtained from rodents is very small, ranging from 1–5μL per tibiofemoral joint in the mouse[32] up to 10–100μL per tibiofemoral joint in the rat[31], and 100–400μL per tibiofemoral joint in the rabbit[75]. Horses and pigs offer a distinct advantage in this regard since volumes of both normal and diseased synovial fluid range from 1.5-3mL per tibiofemoral joint in the pig [76] to 10-12mL per tibiotarsal joint in the horse[77]. In addition to these volume differences of up to four orders of magnitude, the cartilage biology of horses and pigs is very similar to humans[28,33,36–38]. Moreover, these animals are well supported by the FDA as pre-clinical models for osteoarthritis. Lastly, horses suffer from naturally occurring infectious arthritis that requires clinical treatment and rehabilitation protocols similar to that of humans [42,78,79]. Thus, the horse provides an ideal preclinical and clinical model for translational research.

This study offers a powerful alternative to traditional *in vitro* biofilm models to specifically study free-floating biofilm aggregates in physiological fluid. The complexity of host-derived fluids may influence the bacterial phenotype differently than traditional *in vitro* media such as TSB. Therefore, studying bacteria within the context of the infective environment, such as synovial fluid for infectious arthritis or sputum for cystic fibrosis, has the advantage of exploring the bacteria phenotype similar to what is encountered *in vivo*. By utilizing the microenvironment that bacteria encounter upon infection *in vivo*, the robust *ex vivo* model system described in this study offers an important advancement in benchtop biofilm research. Although the limitation of an *ex vivo* study is the lack of pressure from the host immune system or changes that occur within the biofluid during *in vivo* infection, such studies are imperative prior to performing costly long-term *in vivo* studies. By utilizing the equine and porcine model systems described here, we can study the mechanisms by which bacteria form biofilm aggregates in synovial fluid and become tolerant to antimicrobials. The findings from these *in vitro* studies demonstrate a higher degree of model fidelity as the research efforts transition from *in vitro* to *in vivo* model systems. Taken together, we hope that our investigations help advance new therapeutic modalities with the potential to decrease morbidity and mortality associated with infectious arthritis and periprosthetic joint infections.

## Methods

### Bacterial strains

The bacterial strains used in this study were clinical isolates derived from cases of equine septic arthritis collected by the Pennsylvania Animal Diagnostic Laboratory System (PADLS) New Bolton Center Clinical Microbiology Laboratory (*S*. *aureus*, *S*. *zooepidemicus*, *E*. *coli* and *P*. *aeruginosa)*. *In vitro* antimicrobial susceptibility testing and microbial identification was performed using the Sensititre Complete Automated AST System and the equine (Equine EQUIN1F Vet AST Plate) antimicrobial susceptibility panel (Thermo Fisher Scientific, Waltham, MA). Breakpoint-associated minimum inhibitory concentrations (MIC) of each strain are presented in Table 1. Where indicated the laboratory strain of *S*. *aureus*, ATCC25923, was used as a well-characterized control strain. Antimicrobial susceptibility of this strain was determined as described for the clinical isolates. Bacteria were kept in frozen stocks on glycerol at -80°C. Blood agar plates were streaked from frozen stocks and used for *in vitro* experiments for a maximum of 1 week. Overnight cultures were made from the blood agar plates by taking one colony and adding to 30mL of tryptic soy broth (TSB); these cultures were made fresh for each experiment. On the day of an experiment, 100μL of an overnight culture was inoculated into 10mL of fresh TSB and grown to 0.5 McFarland (~3 hours) to ensure the bacteria were in the exponential phase of growth. Concentrations of cultures were confirmed using serial plate dilutions.

### Synovial fluid collection

This study was approved by the Institutional Animal Care and Use Committees of The University of Pennsylvania and the North Carolina State University. Healthy horses free of orthopedic disease and free of medication for 48 hours prior to sampling were used for collection of synovial fluid. Synovial fluid samples were obtained from standing horses sedated with 0.005–0.01 mg/kg detomidine. All horses were well acclimated to standing under sedation for arthrocentesis, which is a short procedure. Both carpi were clipped and aseptically prepped along the dorsal aspect of the joints and 3–4 mL of synovial fluid was extracted from each joint. Following synovial fluid collection, 250mg of amikacin was injected into the joint through the same needle as a preventative measure, as is routinely performed in the clinical setting. Horses were monitored during the procedure and every 12 hours thereafter for 48 hours for signs of discomfort, pain/lameness, swelling at the site of collection, or other adverse effects, none of which were observed. Synovial fluid from both the right and left carpi were pooled among individual horses. Synovial fluid from pigs was collected post-mortem from healthy Yorkshires ~6 months of age. Pigs were part of an unrelated research study of an independent principal investigator at North Carolina State University and were euthanized with 60 mg/kg iv pentobarbital sodium following intramuscular sedation using xylazine (2mg/kg) and ketamine (20 mg/kg) and isofluorane until unconsciousness. Death was confirmed via auscultation. Human synovial fluid was purchased from Lee Biosolutions, Inc. (Maryland Heights, MO). Synovial fluid that was visually cloudy or otherwise abnormal was discarded. Synovial fluid was centrifuged at 1500g for 15 minutes to remove the cellular component and passed through a 40μM cell strainer to remove any large protein aggregates. The samples were stored at -20°C until use in the described experiments. All experiments were performed with four biological replicates (i.e. synovial fluid from four individual horses, humans or pigs).

### Synovial fluid biofilm aggregates and planktonic growth conditions

Planktonic bacteria were grown in tryptic soy broth (TSB). Biofilm aggregates were grown in synovial fluid from the indicated mammalian species. All growth conditions were inoculated with 1x10^6^ CFU/mL[17] of each bacterial strain in a microtiter plate (24-well or 6-well with 500μL or 2mL of media respectively) and incubated overnight (16–18 hours) in a microaerophilic chamber (AnaeroPack-MicroAero Gas Generator, Thermo Fisher Scientific, Waltham, MA) on a shaker at 120rpm at 37°C. Antimicrobial treatments and dispersal treatments were implemented during mid to late exponential phase or 6 hours post-infection (S4 Fig) and added to TSB or synovial fluid containing planktonic or biofilm aggregated bacteria respectively. Macroscopic images were taken with a Nikon D40 camera.

### Confocal microscopy

Bacteria were stained with BacLight Green (Thermo Fisher Scientific, Waltham, MA) prior to inoculation of synovial fluid; after overnight culture in synovial fluid, macroscopic aggregates were gently removed from synovial fluid and washed three times with phosphate buffered saline (PBS). Aggregates were suspended in PBS and stained with wheat germ agglutinin (WGA, Thermo Fisher Scientific, Waltham, MA; 20μg/mL) for 15 min at room temperature in the dark. Supernatant containing WGA was removed and aggregates were stained with 1mL undiluted SYPRO (FilmTracer SYPRO Ruby Biofilm Matrix Stain, Thermo Fisher Scientific, Waltham, MA) for 30 min at room temperature in the dark. Thereafter, stain was removed and aggregates were washed three times with PBS before fixation in 2% paraformaldehyde. Aggregates were kept at 4°C until imaging. Imaging was performed using a Leica SP5 Confocal/Multiphoton Microscope at the PennVet Imaging Core.

### Scanning electron microscopy (SEM)

Scanning electron microscopy images were attained by the staff at the Electron Microscopy Resource Laboratory (EMRL) at the University of Pennsylvania. In brief, aggregates were washed, fixed in glutaraldehyde, dehydrated, gold sputter coated and subsequently imaged with a scanning electron microscope at 3000x with a FEI-Tecnai T12 microscope at the PennMed Imaging Core.

### Antimicrobial treatment

Results from the phenotypic susceptibility testing (Table 1) were used to estimate the MIC (μg/mL) by microbroth dilution of planktonic cultures using the Sensititre Complete Automated AST System and the equine (Equine EQUIN1F Vet AST Plate) antimicrobial susceptibility panel. The vancomycin MIC for *S*. *aureus* (ATCC25923) was 0.5μg/mL as determined by microbroth dilution following CLSI standards. Microtiter wells containing infected synovial fluid (biofilm aggregates) or TSB (planktonic) were treated with antimicrobials at 1×, 10× or 100× the reported MIC of each individual planktonic bacteria during mid to late exponential phase or 6 hours post-infection (S4 Fig). If an MIC was determined to be less than the lowest concentration evaluated, that evaluated concentration was used. For example, the MIC of Amikacin for *S*. *aureus* (ATCC25923) was ≤ 4μg/mL; therefore, bacteria was treated with amikacin at 4μg/mL for 1×MIC, 40μg/mL for 10×MIC, and 400μg/mL for 100×MIC. Antimicrobial treatments were carried out for 8 or 24 hours where indicated under the same growth conditions as the infective period. The infected TSB or synovial fluid was centrifuged at 8000g for 10 min and the supernatant was removed. The bacterial pellet was washed 3x with PBS and resuspended in 1mL of PBS containing 200μg/mL proteinaseK and incubated for 5–10 minutes on a shaker at 120rpm at 37°C to disperse the aggregates for enumeration of bacterial concentration by serial dilutions and plate counting of colony forming units (CFU/mL). This wash and proteinaseK step is critical for appropriate enumeration of bacteria as CFU/mL due to the inability to measure bacterial concentration within the biofilm aggregates. Dastgheyb et al. 2015 first showed the inaccuracy of measuring CFU/mL from intact aggregates and described the ability of proteinaseK to disperse aggregates for accurate enumeration of CFU/mL[17].

### Dispersal treatment

ProteinaseK (200μg/mL), trypsin (200μg/mL), endopeptidase (200μg/mL), DNase (500μg/mL) collagenase type II (750μg/mL), collagenase type IV (750μg/mL), dispase (500μg/mL), DispersinB (100μg/mL), acetylcysteine (8mg/mL), and tissue plasminogen activator or TPA (1mg/mL) were used to test dispersal of biofilm aggregates. All enzymes were purchased from Sigma-Aldrich (St. Louis, MO) apart from DispersinB which was purchased from Kane Biotech (Winnipeg, Canada).The concentration of each enzyme was chosen as the highest concentration that did not exhibit bactericidal effects against planktonic bacteria grown in TSB. Each enzyme was incubated in the infected synovial fluid for 1 hour on a shaker at 120rpm at 37°C. Photographs of the dispersal treatment were taken with a Nikon D40 camera. Dispersal was evaluated by measuring optical density (OD) on a microtiter plate reader (Synergy 2, BioTek Instruments, Inc., Winooski, VT). Optical density was measured as an average of the absorbance (600nm) using a well-mode, or area scanning, method to ensure the entire well was measured. Dispersal was reported as a percentage compared to planktonic bacteria (each bacterial strain was used as its own internal control) in TSB at the same CFU/mL. Specifically, percentage dispersal was calculated as [(OD infected synovial fluid–OD uninfected synovial fluid)/(OD infected TSB–OD uninfected TSB)] X 100. This method was developed based on measurements and calculations of platelet aggregation[80–82]. Bacterial viability (CFU/mL) was measured post-dispersal and compared to the no treatment control to ensure that enzymatic treatment did not induce cell death. After 1 hour of dispersal, enzymatically treated synovial fluid samples containing biofilm aggregates were challenged with amikacin at 10× MIC for 8 hours. Bacterial viability (CFU/mL) was assessed post-dispersal and antimicrobial challenge using serial dilutions and colony counting as described above.

### Statistics

Data was analyzed using 1-way non-parametric (Kruskal-Wallis Test) or 1-way/2-way ANOVA where applicable with Tukey's post hoc tests. Correlations were calculated using Spearman correlation coefficient. Analysis was performed using JMP Pro 11.0 software (SAS Institute Inc., Cary, NC). All graphs were generated using GraphPad Prism (GraphPad Software Inc., La Jolla California USA). For all comparisons, p<0.05 was considered statistically significant.

## Supporting information

S1 Fig Time dependent growth and biofilm aggregate formation in equine synovial fluid. Equine synovial fluid was infected at 1x10^6^ CFU/mL with *S*. *aureus* (ATCC25923) and incubated overnight at 37°C in a microaerophilic chamber on a shaker at 120rpm to mimic the joint environment. (A) *S*. *aureus* growth in synovial fluid over time was measured by treating synovial fluid with proteinaseK (20μg/mL) to disperse aggregates, followed by serial dilutions and plate counting for CFU/mL. (B) Biofilm aggregate formation was photographed at the same time as bacterial load determination.
(TIF)

S2 Fig Planktonic bacteria are inhibited or killed by several different classes of antimicrobials. *S*. *aureus* (ATCC25923) was grown planktonically in TSB for 6 hours and challenged with a panel of different antimicrobials from several drug classes at 100× the minimum inhibitory concentration (MIC) as determined by in vitro antimicrobial susceptibility testing (Table 1).
(TIF)

S3 Fig Hyaluronidase pre-treatment but not post-treatment of synovial fluid prevents biofilm aggregate formation and development of antimicrobial tolerance. (A) Equine synovial fluid was either left untreated or pre-treated with hyaluronidase (1mg/mL) prior to infection with *S*. *aureus* (ATCC25923). Bacteria were added, incubated for 16 hours and either left untreated or post-treated with hyaluronidase (1mg/mL) for 1 hour. Thereafter, amikacin was added at 10× MIC (40μg/mL), incubated for 8 hours, and log CFU/mL was measured with serial dilutions and colony counting. Bars are means and standard deviations of four biological replicates (n = 4), and significant differences (p<0.05) as determined by ANOVA with Tukey post-hoc are indicated by differing letters.
(TIF)

S4 Fig Enzymatic treatment of synovial fluid containing biofilm aggregates does not alter bacterial load >1 log CFU/mL. Equine synovial fluid containing *S*. *aureus* (ATCC25923) biofilm aggregates were treated with: hyaluronidase (1mg/mL), proteinaseK (200μg/mL), trypsin (200μg/mL), endopeptidase or LysC (200μg/mL), DNase (500μg/mL), collagenase type II (750μg/mL), collagenase type IV (750μg/mL), dispase (500μg/mL), DispersinB (1mg/mL), acetylcysteine (8mg/mL) or tissue plasminogen activator or TPA (1mg/mL). Bacterial load (log CFU/mL) was measured with serial dilutions and colony counting 9 hours post-enzymatic treatment. Bars are means and standard deviations of four biological replicates (n = 4), and significant differences (p<0.05) as determined by ANOVA with Tukey post-hoc are indicated by differing letters.
(TIF)
