# Supplementary figures and images for: Equine or porcine synovial fluid as a novel ex vivo model for the study of bacterial free-floating biofilms that form in human joint infections
Source: PLoS One. 2019 Aug 15;14(8):e0221012. doi: 10.1371/journal.pone.0221012 (PMC6695105; doi:10.1371/journal.pone.0221012)

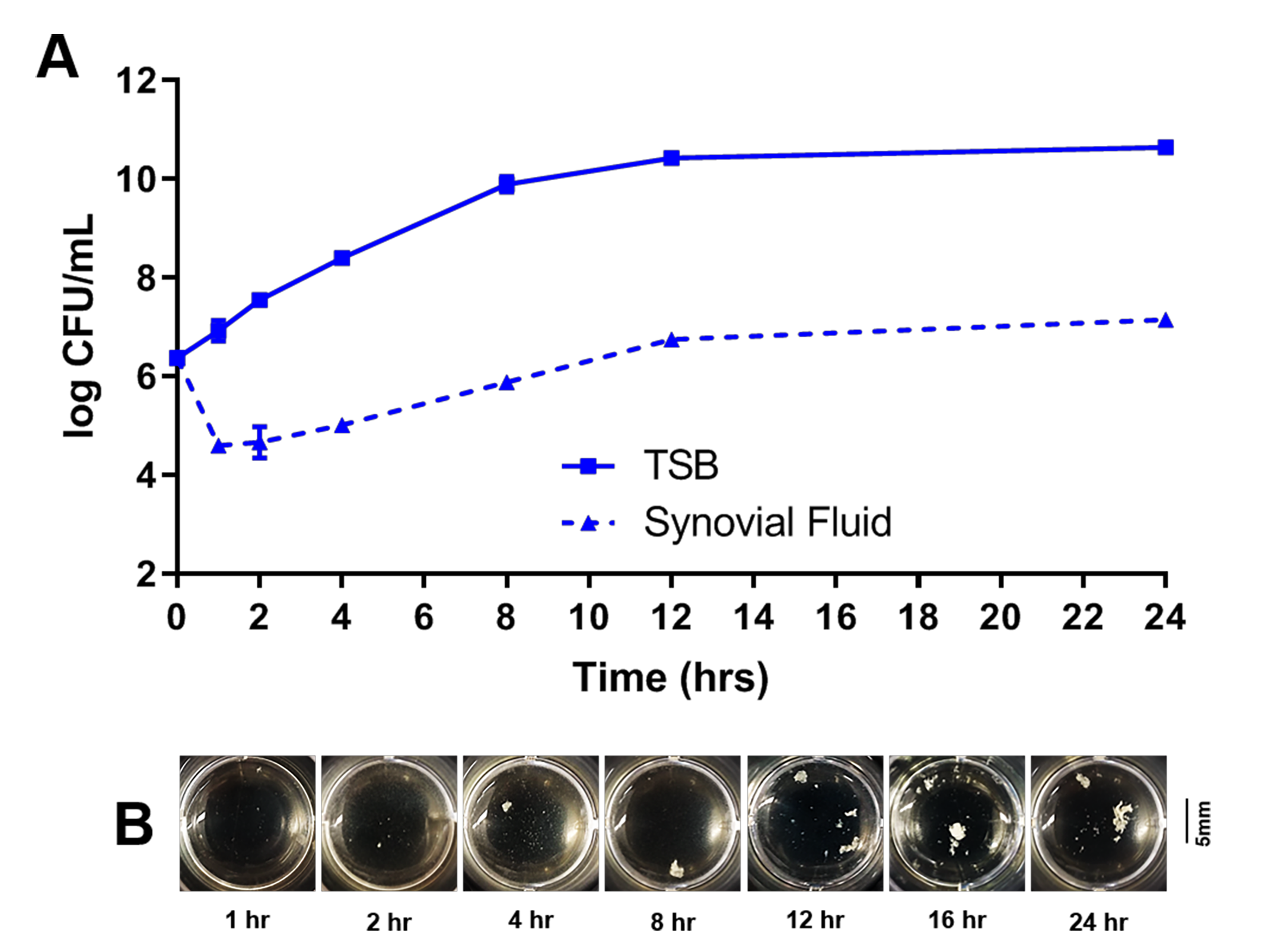

Supplement: S1 Fig — Equine synovial fluid was infected at 1x10^6^ CFU/mL with S. aureus (ATCC25923) and incubated overnight at 37°C in a microaerophilic chamber on a shaker at 120rpm to mimic the joint environment. (A) S. aureus growth in synovial fluid over time was measured by treating synovial fluid with proteinaseK (20μg/mL) to disperse aggregates, followed by serial dilutions and plate counting for CFU/mL. (B) Biofilm aggregate formation was photographed at the same time as bacterial load determination. (TIF) [file pone.0221012.s001.tif]

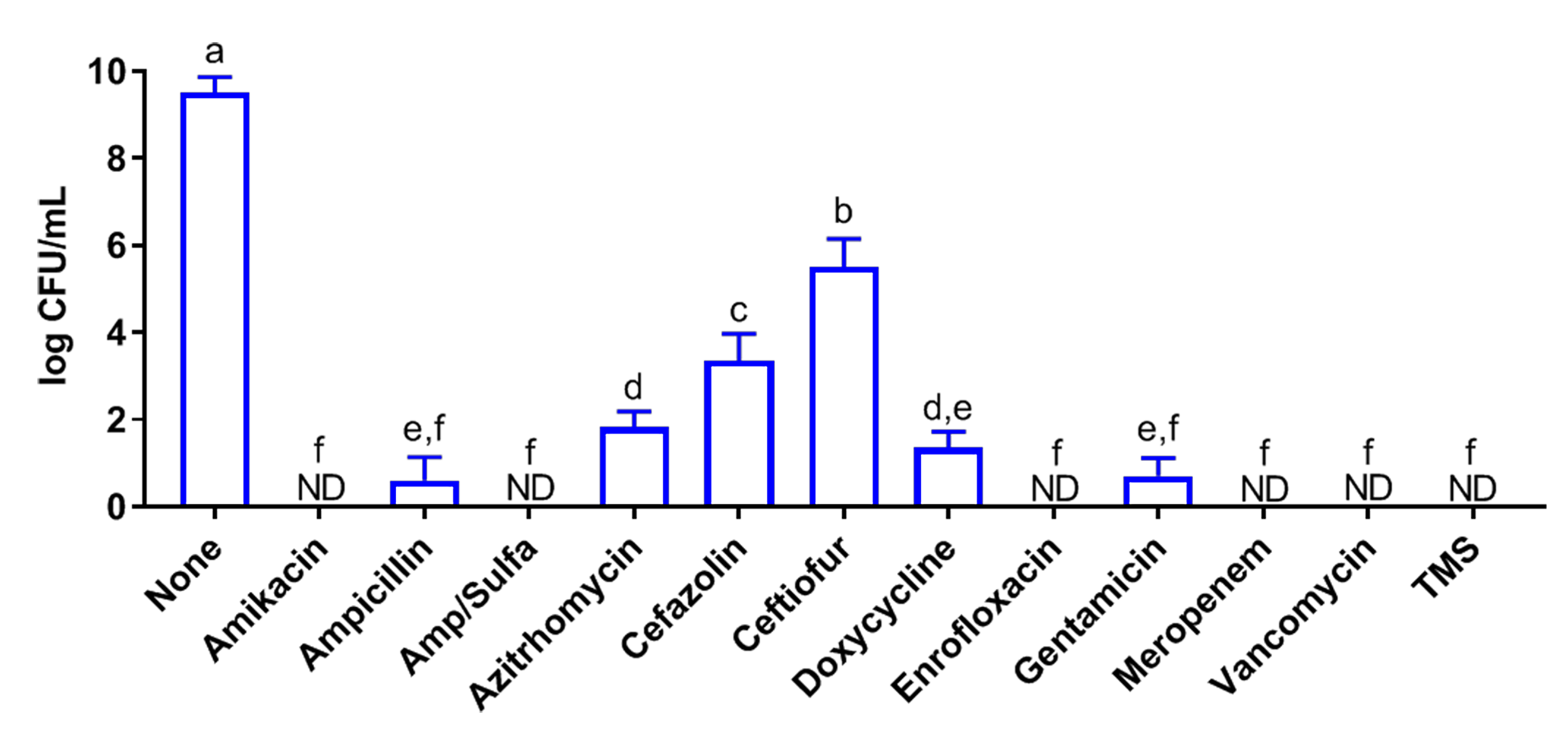

Supplement: S2 Fig — S. aureus (ATCC25923) was grown planktonically in TSB for 6 hours and challenged with a panel of different antimicrobials from several drug classes at 100× the minimum inhibitory concentration (MIC) as determined by in vitro antimicrobial susceptibility testing (Table 1). (TIF) [file pone.0221012.s002.tif]

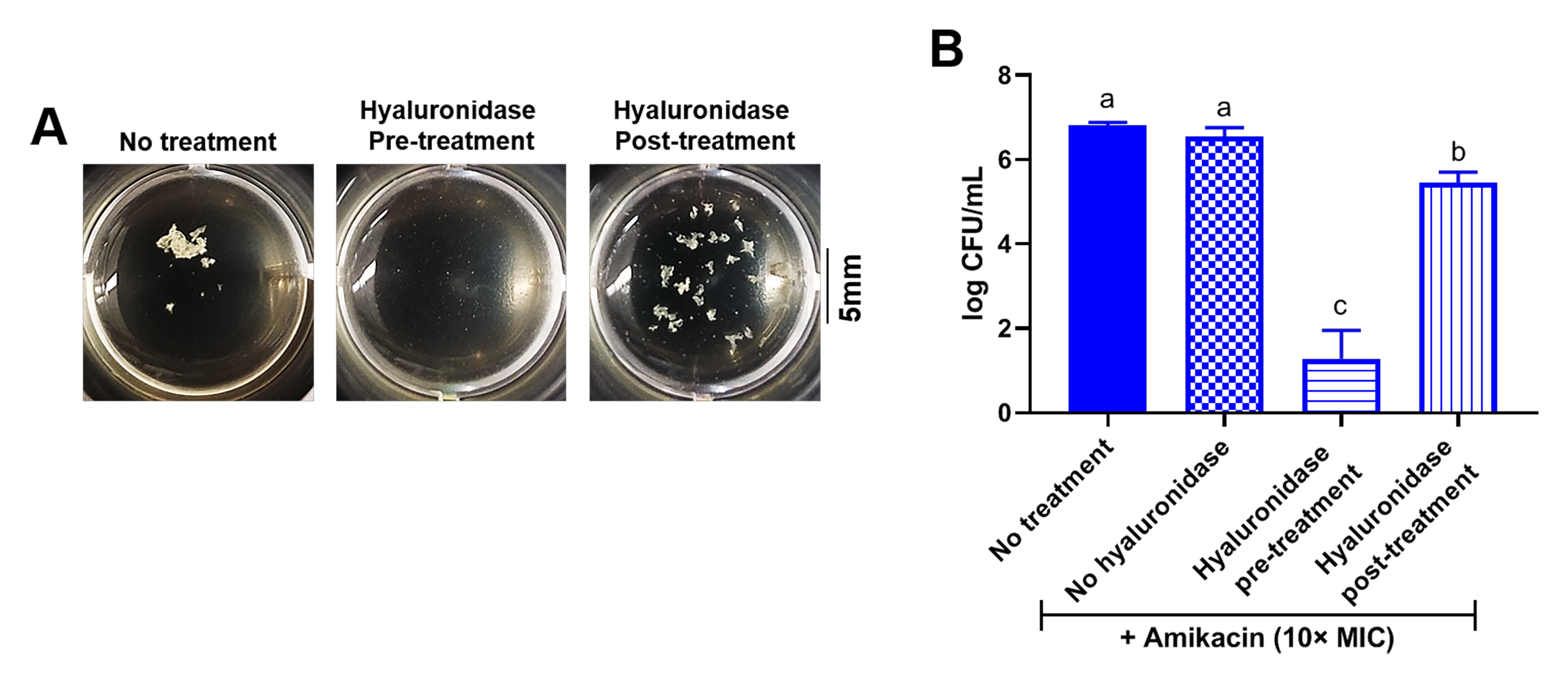

Supplement: S3 Fig — (A) Equine synovial fluid was either left untreated or pre-treated with hyaluronidase (1mg/mL) prior to infection with S. aureus (ATCC25923). Bacteria were added, incubated for 16 hours and either left untreated or post-treated with hyaluronidase (1mg/mL) for 1 hour. Thereafter, amikacin was added at 10× MIC (40μg/mL), incubated for 8 hours, and log CFU/mL was measured with serial dilutions and colony counting. Bars are means and standard deviations of four biological replicates (n = 4), and significant differences (p<0.05) as determined by ANOVA with Tukey post-hoc are indicated by differing letters. (TIF) [file pone.0221012.s003.tif]

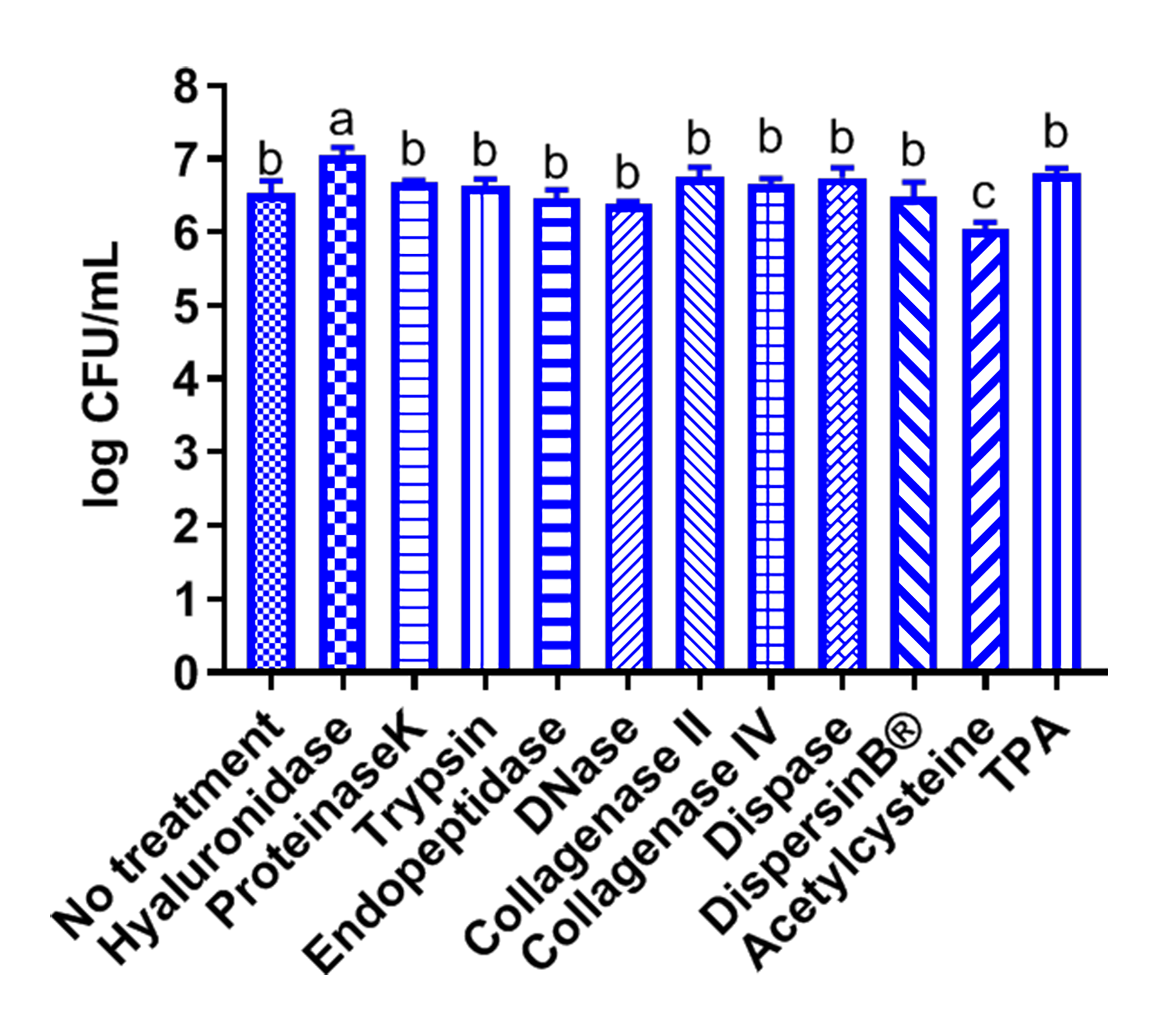

Supplement: S4 Fig — Equine synovial fluid containing S. aureus (ATCC25923) biofilm aggregates were treated with: hyaluronidase (1mg/mL), proteinaseK (200μg/mL), trypsin (200μg/mL), endopeptidase or LysC (200μg/mL), DNase (500μg/mL), collagenase type II (750μg/mL), collagenase type IV (750μg/mL), dispase (500μg/mL), DispersinB (1mg/mL), acetylcysteine (8mg/mL) or tissue plasminogen activator or TPA (1mg/mL). Bacterial load (log CFU/mL) was measured with serial dilutions and colony counting 9 hours post-enzymatic treatment. Bars are means and standard deviations of four biological replicates (n = 4), and significant differences (p<0.05) as determined by ANOVA with Tukey post-hoc are indicated by differing letters. (TIF) [file pone.0221012.s004.tif]
